# The safety and efficacy of astragalus for treating diabetic foot ulcers: A protocol for systematic review and meta-analysis

**DOI:** 10.1097/MD.0000000000024082

**Published:** 2021-01-08

**Authors:** Donghao Liu, Yanmei Zhong, Hang Yan, Yuanzhang Hu, Yuzhu Chen, Yi Zhou

**Affiliations:** a School of Basic Medical Sciences; b School of Medical Information Engineering, Chengdu University of Traditional Chinese Medicine; c Affiliated Hospital of Chengdu University of Traditional Chinese Medicine, Chengdu, Sichuan, P. R. China.

**Keywords:** Astragalus, diabetic foot ulcers, effectiveness and safety, meta-analysis

## Abstract

**Background:**

Diabetic foot ulcers are the most common complication among diabetic patients, which may put the patients in a great danger of amputation. Astragalus as a Chinese herbal medicine has been reported in many publications that it has an efficacy toward diabetic foot ulcers. However, the systematic review and meta-analysis of its efficacy and safety are still absent. Therefore, we aim to evaluate the effectiveness and safety of Astragalus for diabetic foot ulcers.

**Methods:**

The following databases will be searched from January 1st, 2010 to September 2020: The Cochrane Library, Pubmed, EMBASE, Web of Science, China National Knowledge Infrastructure, and Wanfang Data. All the English and Chinese publications will be searched without any restriction of countries. Data will be extracted by 2 reviewers independently. RevMan 5.4.1. will be used to perform analysis and synthesis of data.

**Results:**

This meta-analysis of randomized controlled trials will evaluate the efficacy and safety of Astragalus for diabetic foot ulcers during the past 10 years.

**Conclusion:**

This study will provide an evidence to judge whether Astragalus is effective and safe for diabetic patients with foot ulcers.

**INPLASY registration number:**

Inplasy protocol 2020110059. (doi:10.37766/inplasy2020.11.00596).

## 1 Introduction

Diabetic foot ulcers (DFU) are the most common complication in diabetic patients.^[1]^ The annual incidence of foot ulceration among people with diabetes is about 9.26 million. About 80% of nontraumatic lower-extremity amputations are presented with foot ulceration. Furthermore, diabetic patients who suffer from foot ulcers encounter financial burden on health 5 times higher than those without foot ulcers.^[2]^ Astragalus as a traditional Chinese herbal medicine has been reported in many publications that it can treat DFU by increasing the number of body fibroblasts, promote cell proliferation, reducing the activity of galactosidase in ulcers, improving cell aging, and delaying cell aging.^[3–6]^ However, there is currently no systematic review of the safety and effectiveness of Astragalus in the treatment of DFU. Therefore, we propose a protocol for a systematic review to evaluate the effectiveness and safety of in the treatment of DFU.

## 2 Methods

### 2.1 Study registration

The protocol for this systematic review has been registered on INPLASY (Inplasy protocol 2020110059. doi:10.37766/inplasy2020.11.00596) and is available in full on the inplasy.com.

### 2.2 Inclusion criteria for study selection

#### 2.2.1 Types of studies

Only randomized controlled trials can be included. Observation studies, animal experiment, case report, review, and meta-analysis are excluded. All the English and Chinese publications will be searched without any restriction of countries.

#### 2.2.2 Participants/population

The studies will involve adult participants who meet the diagnostic criteria for DFU. All eligible study participants will be included in this review, regardless of gender, race or occupation. However, participants who are pregnant, breastfeeding, menstruating or suffering from other serious illnesses will be excluded.

#### 2.2.3 Types of interventions

We will include all randomized controlled trials which uses Astragalus alone (eg, oral administration, Astragalus injection, and topical use) or in combination with other medical interventions.

#### 2.2.4 Comparator

The control group will be treated with commonly used western medicine (such as Buflomedil, Urokinase, Ozagrel, Recombinant Human Growth Hormone, DL-Thioctic acid, etc), placebo or no intervention measures.

#### 2.2.5 Types of outcome measures

The primary outcome will be the rate of healing based on the Wagner classification. Additional outcomes will be recurrence rate, size of the ulcers, and amputation rate, clinical effective rate, and adverse event rate.

### 2.3 Data source

The following databases will be searched from 1st January 2010 to September 2020: The Cochrane Library, Pubmed, EMBASE, Web of Science, China National Knowledge Infrastructure, and Wanfang Data. All the English and Chinese publications be searched without any restriction of countries.

### 2.4 Search strategy and study selection

#### 2.4.1 Search strategy

Different Search strategies will be applied according to the requirements of different databases. The specific search strategy for PubMed is revealed in the Table 1.

**Table 1:** Specific search strategy for PubMed.

No	Search terms
#1	diabetic foot ulcer^∗^
#2	foot ulcer, diabetic
#3	ulcer, diabetic foot
#4	plantar ulcer^∗^, diabetes
#5	#1OR #2 OR #O3 #04
#6	Astragalus Plants
#7	Astragalus
#8	Radix Astragali
#9	huang qi
#10	#6OR #7 OR #O8 #09
#11	randomized clinical trails
#13	RCT
#14	# 11OR #12

#### 2.4.2 Study selection

**Figure 1:**
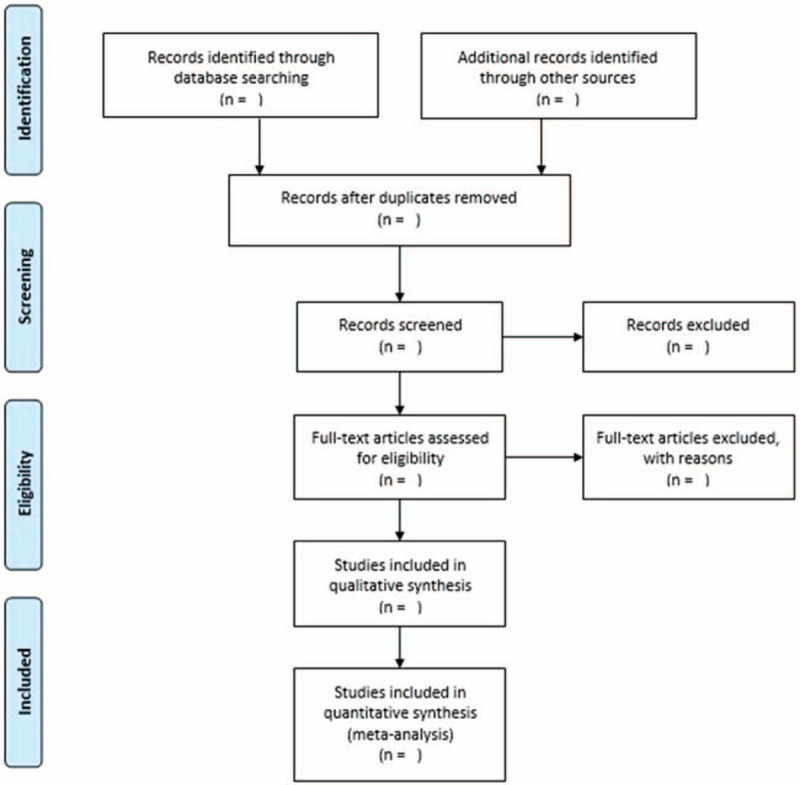
Flow diagram of the study selection process.

Removal of duplication and documents management will be conducted by EndNote X9 software. Two authors will independently eliminate relatively unrelated literature by scanning titles, abstract. When there is dispute, the 2 reviewers will read the full text. Any disagreement will be solved by discussion. If there is missing information, we will contact the corresponding author. Diagram of the study selection process is shown in the Figure 1.

### 2.5 Data extraction and analysis

#### 2.5.1 Data extraction

Data will be extracted by 2 reviewers independently. Any disagreement will be resolved by discussion until consensus is reached or by consulting a third author. The following data will be extracted: general information: author, year of publication, country where the study was conducted; participant characteristics, including age, gender, time since ulcerate, classification of the ulcers according to Wagner classification, total number of people included in the study; Intervention details: doses of Astragalus, the time of application and administration form.

#### 2.5.2 Strategy of data synthesis

The analysis and synthesis of data will be conducted by RevMan 5.4.1. The random-effects model and the fixed-effects model will be applied, when *I*^2^ is more than 50% and *I*^2^ is less than 50%, respectively. Risk ratio with 95% confidence interval (CI) will be used to determine dichotomous data and weighted mean differences (with 95% CI) or standardized mean differences (95%) CI will be used to analyze the continuous data.

#### 2.5.3 Risk of bias assessment

The methodological quality of randomized controlled trials will be assessed by Cochrane risk of bias. It includes generation of the allocation sequence; concealment of the allocation sequence; blinding; attrition and exclusions; other generic sources of bias; biases specific to the trial design (such as crossover or cluster randomized trials); and biases that might be specific to a clinical specialty. Any disagreement will be addressed by discussion among authors.

#### 2.5.4 Analysis of subgroups

Subgroup analysis will be implemented according to administration types, and different outcome measures when there is substantial heterogeneity.

#### 2.5.5 Sensibility analysis

If necessary, a sensitivity analysis will be performed.

#### 2.5.6 Grading the quality of evidence

The Guidelines for Evaluation, Development, and Evaluation of Recommendations will be used to classify the quality of the tests as very low, low, moderate, or high. The risk of bias, heterogeneity, and publication bias will be also assessed.

### 2.6 Ethics

This is a protocol, so ethical approval is not necessary.

## 3 Discussion

DFU is one of serious complications of diabetes, which can be attributed to neurological disorders, peripheral arterial disease, and immuno-suppression.^[7–10]^ According to international diabetes federation, the annual incidence of foot ulcers among people with diabetes is about 2%. About 1% of diabetics have, at some stage, lower-limb amputation.^[11,12]^ And 5% Patients with DFU will undergo a major amputation and 20% to 30% patients with DFU have a minor amputation.^[13]^ Astragalus as a traditional Chinese herbal medicine, has been widely used in China for treating DFU.^[5,14–16]^ This systematic review will be the first to assess the efficacy and safety of Astragalus for DFU, which may facilitate the clinical practice of Astragalus.

## Author contributions

**Conceptualization:** Donghao Liu, Yanmei Zhong, Yi Zhou.

**Data curation:** Hang Yan.

**Funding acquisition:** Yi Zhou.

**Methodology:** Donghao Liu, Yanmei Zhong.

**Project administration:** Donghao Liu, Yanmei Zhong, Yi Zhou.

**Resources:** Yuzhu Chen.

**Software:** Yuanzhang Hu.

**Validation:** Yi Zhou.

**Writing – original draft:** Donghao Liu, Yanmei Zhong.

**Writing – review & editing:** Donghao Liu, Yanmei Zhong.
